# Cognitive biases as interrupters in evidence based practice decision-making

**DOI:** 10.5195/jmla.2025.2209

**Published:** 2025-10-23

**Authors:** Jonathan D. Eldredge, Deirdre A. Hill

**Affiliations:** 1 jeldredge@salud.unm.edu, Professor, Health Sciences Library and Informatics Center. Professor, Department of Family & Community Medicine, School of Medicine. Professor, College of Population Health University of New Mexico, Albuquerque, NM; 2 DAHill@salud.unm.edu, Associate Professor, School of Medicine, University of New Mexico, Albuquerque, NM

**Keywords:** Decision Making, Cognitive Bias, Evidence Based Practice, Leadership, Group Processes, Medical Library Association, Health Sciences Librarianship, Health Information Professionals, Informaticists

## Abstract

**Objectives:**

To identify the most frequently-observed forms of cognitive bias among Health Information Professionals (HIPs) during decision-making processes. To determine if number of years in the profession influences the types of cognitive biases perceived in others' decisions.

**Method:**

This cross-sectional study invited participation of 498 elected and appointed leaders at the national, caucus, and chapter levels of the Medical Library Association. The 149 participants (32%) were presented with 24 cognitive biases often associated with expected decision-making contexts among HIPs.

**Results:**

The most frequently observed forms of cognitive bias in decision-making situations were: Status Quo, Sunk Costs, Novelty, Professionology, Authority, Worst-Case Scenario, and Group Think. Four of these overlapped with a previous 2007 study. Results were analyzed by length of years in the profession. Four forms of cognitive bias showed statistically significant differences in frequency by years in the profession: Authority, Naïve Realism, Overconfidence, and Status quo forms of cognitive bias.

**Discussion:**

This study identified commonly observed cognitive biases that interrupt decision-making processes. These results provide a first step toward developing solutions. Mitigation strategies for the seven most common forms of identified cognitive bias are explored with more general recommendations for all forms of cognitive bias. This study should guide the profession on the most commonly-perceived forms of cognitive bias occurring in decision-making environments with an eye upon possible mitigation strategies.

## INTRODUCTION

For more than two decades the health professions have used the Evidence Based Practice (EBP) approach to making sound decisions. While EBP has proven itself to a be a durable framework, practitioners sometimes note that as they progress through the EBP steps of question formulation, searching, critical appraisal, and deciding–something towards the end of the process goes awry [1-2]. Since the purpose of EBP hinges on making decisions upon the best available evidence, any investigation to improve this process will be crucial [3]. Cognitive biases appear to interrupt the EBP process between evidence appraisal and the final “mystery decision”[4].

Cognitive biases are well-known interrupters in making decisions. Cognitive biases are everyday human tendencies to either fail to perceive a situation correctly or to think clearly about those situations when making a decision. Evolutionary psychologists, [5-8] biologists, [9] and economists [10] have postulated that cognitive biases were essential for our survival in a time when our species was first emerging during an era when we were not the apex predators. Cognitive biases tend to emerge most often when people are confronted with ambiguous, complex, or large amounts of information [11-12]. To cite only two common examples, when confronted with a long series of complex information, people tend to lock-in on either information provided early in the sequence, known as Primacy Bias, [13] or late in the sequence, known as Recency Bias[14]. Cognitive biases are largely unavoidable and everyone succumbs to them in varying degrees. Most importantly, people typically are unaware of their own cognitive susceptibilities even if they can easily spot others' susceptibilities. [15-17].

Researchers have recognized and studied cognitive biases for the past century, identifying over 170 cognitive biases in decision-making contexts. While many of these cognitive biases might contribute to precursors to decision-making processes, only an estimated 20-30 cognitive biases directly affect the kinds of contexts of decisions made ordinarily by health information professionals (HIPs). In this study, HIPs are defined as informaticists, health sciences librarians, information scientists, informationists, or archivists.

HIPs make numerous decisions on a daily basis in both individual tasks and in group contexts. One US study on everyday decision-making conducted in 2007 presented health sciences librarians with a list of 21 cognitive biases with definitions and asked respondents to indicate which three (3) they had witnessed most often among their HIP colleagues when engaged in decision-making. **Supplementary Table 1** in the online appendix lists the 135 respondents' most commonly observed cognitive biases [18–19]. A search of the literature since 2007 has not turned up any similar empirical research study on cognitive biases involving HIPs.

The present study updated and aimed to improve upon the methodological rigor found in the 2007 study. The authors began this study with the following hypotheses:

*Hypothesis 1*: HIP leaders would identify only some of the same cognitive biases among fellow HIPs compared to 2007 due to the changeability over time of identified forms of cognitive bias observed in other professionals;

*Hypothesis 2*: HIP leaders with more experience in the field would markedly differ in the forms of cognitive biases that they would observe compared to leaders with fewer years of experience in the profession.

### Methods

This cross-sectional study measured the prevalence of certain forms of cognitive bias observed by leaders in contexts when other HIPs were making decisions. The online appendix includes a Detailed Methods Description that recounts the lengthy, iterative processes of creating a suitable inventory of cognitive biases and then later testing the survey instrument. This Methods section provides some most immediately relevant details. The authors received IRB approval (HRRC 24-168) from the University of New Mexico Human Research Review Committee on April 11, 2024.

#### Leaders List

Medical Library Association (MLA) leaders on the national level were defined as all elected officials, editors, and all chairs and members appointed to national level committees. At the caucus and chapter level, leaders were defined as all elected officers and appointed committee chairs. Names and email addresses were obtained from rosters to create a list of 499 leaders generated for the MLA Research Agenda [20],

#### Deployment

On Monday May 13, the final REDCap version of the cognitive bias survey was launched to 498 leaders in the Medical Library Association, excluding one as a recusal for the first author. A total of 26 (5%) of the intended recipients could not be reached, mostly due to returned undeliverable messages; some had retired, left their organization for unstated reasons, were on sabbatical, or on parental leave. These undeliverable messages resulted in 472 potential recipients. Reminders were emailed to all 498 leaders May 17, 23, and 27 and on June 16, 2024.

Participants who consented were presented with 24 forms of cognitive bias with definitions for each. The directions in Part 1 stated: “Read and reflect upon each of the forms of cognitive bias below. Recall instances involving fellow health information professionals having their decisions interrupted by their cognitive biases.” Each time someone opened the REDCap survey, they were presented with a new randomized sequence of cognitive bias to prevent either primacy, [21] recency bias, [22] or response order bias [23-24] from interfering with survey participants' voting preferences. In Part 2 participants were asked to “Please select up to five (5) forms of cognitive bias that you have observed most often in health information professions colleagues.” The survey parameters allowed as few as one yet no more than five choices in Part 2.

Part 3 asked participants: “Your role(s) in MLA (check all that apply).” The results in **Supplementary Table 2** in the online appendix appear to be roughly proportionate to the total numbers of leaders filling these respective roles in MLA. Part 4 asked participants:

How many years have you been an employed health information professional since receiving your terminal professional degree? A terminal degree might be your masters in information science or MLS degree, or, an informatics certificate; or, it otherwise might be a fellowship beyond the MD or PhD.

Part 5 (Optional open-ended question) asked:

Do you have any experiences with cognitive biases disrupting decision making that you would like to share? Please list the name of the specific cognitive bias along with your story. Please exclude any and all identifying information.

The statistician co-author analyzed the descriptive results.

## RESULTS

**Figure 1:**
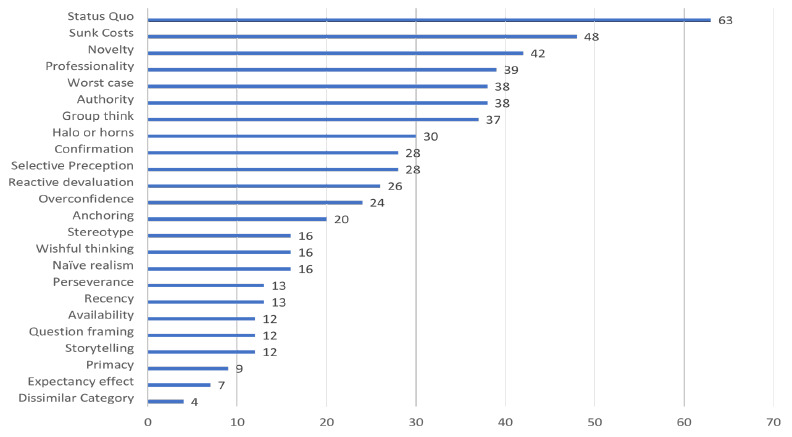
Main Results

The present study involved a secure, anonymous survey delivered through REDCap to MLA 498 leaders to learn what forms of cognitive bias these leaders perceived to be most responsible for interfering with decisions made by colleagues in our profession. These MLA leaders were likely to have a broad perspective and to have observed decision-making in varied contexts. A total of 149 MLA leaders submitted viable cognitive bias surveys, a response rate of 32%. **Supplementary Table 2** in the online appendix indicates that a proportionate number of types of leaders participated in this study. **Figure 1** graphically portrays the ranked order main results while **Table 1** displays them numerically. The top-ranked forms of cognitive bias were: Status Quo, Sunk Costs, Novelty, Professionology, Authority, Worst Case Scenario, and Group Think.

**Table 1:** Most Commonly Observed Cognitive Biases among HIPs in 2007

	Total (N=149 respondants)
Status quo, n (%)	63 (.42.3%)
Sunk Costs, n (%)	48 (32.2.%)
Novelty, n (%)	42 (.28.2%)
Professionology, n (%)	39 (.26.2%)
Authority, n (%)	38 (25.5.%)
Worst case, n (%)	38 (2.5.5%)
Group think, n (%)	37 (24.8.%)
Halo or horns, n (%)	30 (20.1.%)
Selective Preception, n (%)	28 (18.8.%)
Confirmation, n (%)	28 (18.8%)
Reactive devaluation, n (%)	26 (.17.4%)
Overconfidence, n (%)	24 (16.1%)
Anchoring, n (%)	20 (13.4%)
Naïve realism, n (%)	16 (10.7%)
Wishful thinking, n (%)	16 (.10.7%)
Stereotype, n (%)	16 (10.7%)
Recency, n (%)	13 (8.7.%)
Perseverance, n (%)	13 (8.7.%)
Storytelling, n (%)	12 (8.1.%)
Question framing, n (%)	12 (.8.1%)
Availability, n (%)	12 (8.1.%)
Primacy, n (%)	9 (.6.0%)
Expectancy effect, n (%)	7 (.4.7%)
Dissimilar Category, n (%)	4 (2.7%)

Hypothesis 2 stated that “HIP leaders with more experience in the field would markedly differ in the forms of cognitive biases that they would observe compared to colleagues with less experience as HIPs.” **Table 2** presents a nuanced result for Hypothesis 2 regarding number of years as HIPs. Significant differences (p value <= .05) for years in the profession were determined using a chi-square with a Fisher's exact test: Authority, Naïve Realism, Overconfidence, and Status Quo forms of cognitive bias. The two groups did not differ in their frequency in mentioning Novelty bias.

**Table 2:** Years in Profession

	Years in Profession	
	1-14 (N=61)	>14 (N=59)
**Status quo**, n (%)	36 (59.0%)	24 (40.7%)
**Sunk Costs**, n (%)	23 (37.7%)	24 (40.7%)
**Novelty**, n (%)	21 (34.4%)	20 (33.9%)
**Professionology** n (%)	22 (36.0%)	15 (25.4%)
**Authority**, n (%)	13 (21.3%)	23 (38.9%)
**Worst case**, n (%)	17 (27.9%)	20 (33.9%)
**Group think**, n (%)	18 (29.5%)	19 (32.2%)
**Halo or horns**, n (%)	17 (27.9%)	12 (20.3%)
**Selective Preception**, n (%)	14 (23.0%)	14 (23.7%)
**Confirmation**, n (%)	16 (26.2%)	11 (18.6%)
**Reactive devaluation**, n (%)	15 (24.5%)	10 (16.9%)
**Overconfidence**, n (%)	8 (13.1%)	16 (27.1%)
**Anchoring**	8 (13.1%)	12 (20.3%)
**Naïve realism**, n (%)	12 (19.7%)	4 (6.8%)
**Wishful thinking**, n (%)	9 (14.8%)	7 (11.9%)
**Stereotype**, n (%)	9 (14.8%)	7 (11.9%)
**Recency**, n (%)	7 (11.5%)	6 (10.2%)
**Perseverance**, n (%)	9 (14.8%)	4 (6.8%)
**Storytelling**, n (%)	6 (9.8%)	5 (8.5%)
**Question framing**, n (%)	4 (6.7%)	8 (13.6%)
**Availability**, n (%)	5 (8.2%)	7 (11.9%)
**Primacy**, n (%)	3 (4.9%)	6 (10.2%)
**Expectancy effect**, n (%)	4 (6.7%)	3 (5.1%)
**Dissimilar Category**, n (%)	1 (1.6%)	3 (5.1%)

## DISCUSSION

This study sought to identify MLA leaders' most commonly-observed cognitive biases among other Health Information Professionals (HIPs) A comparison of **Table 1** and **Figure 1** with **Supplementary Table 1** in the online appendix that summarizes the 2007 study confirm Hypothesis 1 that four forms of cognitive bias were the same between the 2007 and 2024 studies: Professionology, Status Quo, Authority, and Group Think. Nevertheless, the relative rank orders are different between studies.

These 2024 results suggest several themes. The two highest ranked forms of cognitive bias, Status Quo and Sunk Costs, reflect disapproval in the minds of observers with wanting to preserve existing practices. This presents a paradox since HIPs uphold an altruistic mission to preserve an accurate and permanent record as a means to lend integrity to the evidence base. On a more pragmatic level, our profession resembles other professions in having developed time-tested practices through trial and error. The paradox continues when contemplating the third-ranked cognitive bias of Novelty, which seems diametrically opposed to the top two-ranked forms. One is immediately struck by the apparent inconsistency between the two top-ranked Status Quo and Sunk Cost biases with the diametrically-opposed third, Novelty bias. This contradiction might speak to the human condition of experiencing conflict when making choices between established approaches and the need to take possible risks on a new course of action to foster possible progress. HIPs similarly might have to navigate between retaining an accurate record of the past while serving in their frequent expected roles as early adapters of information technology within their organizations. The two years-in-the-profession groups also did not differ statistically in their frequency in mentioning of Novelty bias.

### Mitigation Strategies

Seven forms of cognitive bias emerged from this cross-sectional study as most commonly observed among fellow HIPs. These seven forms of cognitive bias appear below in rank order of most- to less-mentioned forms with suggested strategies for mitigation. **Table 3** summarizes these mitigation strategies concisely.

**Table 3:** Top-Ranked Cognitive Biases and Their Possible Mitigation

Rank	Cognitive Bias	Description	Possible Mitigation Strategies
1	Status Quo	*Desiring to keep conditions relatively similar to one's present state and therefore predictable.*	Aggressively seek out information that negates any pending decision Compose heterogenous decision making teams with members with diverse backgrounds Assign separate teams with same parallel task of developing their own recommendations Inform decision makers that their decision will be reviewed by an external expert
2	Sunk Costs	*To place undue emphasis on retaining an existing resource when making a decision when another unowned resource might be superior.*	Query decision makers with their past of current economic hardship that might exaggerate their frugalness in weighing the financial implications of an organizational rather than an individual decision Parent organization delegates reviewing the decision to person(s) with no prior connection to the original decision
3	Novelty	*The initial fascination and enthusiasm for a new technology or an innovation that does not yet have the needed evidence to support its adoption.*	Recognizing that early glowing reports of new innovations often have not been rigorously or extensively tested to prove their superiority
4	Professionology	*Viewing a situation through the shared perceptions of one's profession rather than by taking a broader perspective.*	Increase opportunities for interactions or collaborations between members of different professions Encourage networking and friendships between members of different professions Encourage an open-minded engagement with information that runs counter to the profession's attitudes
5	Authority	*Deferring to an expert or other authority figure disproportionate to the extent of their expertise; or, the range of their authority on the subject*.	Colleagues need to scrutinize any decisions that appear to be outside the range of expertise of any decision makers Pause any decision long enough for others to apply their critical analysis to the pending decision. Encourage those lower in any organizational hierarchy to question any suspect decisions
6	Worst Case Scenario	*Emphasizing or exaggerating those possible negative outcomes disproportionate to all possible outcomes*.	Encourage colleagues to visualize and articulate their feared worst-case scenario in graphic detail, which paradoxically often changes their perspective List the best case and worst-case scenarios side-by-side to appreciate the full range of possibilities instead of only the worst possible outcome Add more experienced colleagues to the decision-making group to offer a more experienced range of possibilities to the deliberations
7	Group Think	*Believing in the autonomy of a group, stereotyping of those outside the group, self-censoring, censoring of dissenters, maintaining the illusion of unanimity, and enforcing a group “consensus” viewpoint.*	Appoint 1-2 group members with responsibility to argue against the dominant opinions in the group Leader should embrace minority viewpoints in the group as a counterbalance Leaders explicitly recommend to all group members to scrutinize any pending decision critically Encourage all group members to adhere to a scientific mindset when reviewing possible decisions Assemble decision-making teams with members known to hold different views on the decision

### Status Quo

*Desiring to keep conditions relatively similar to one's present state and therefore predictable.*

A total of 42% (*n* = 63) of the respondents selected Status Quo bias. A number of studies have sought to better understand Status Quo bias by analyzing possible psychological or organizational patterns leading to this dysfunction [25-28]. **Table 3** summarizes some concrete methods for countering Status Quo bias based on several studies [29-33]. Status Quo bias presents many in our field with a dilemma in that we are responsible for the integrity and preservation of the information, which might habitually contradict some otherwise reasonable proposed changes.

### Sunk Costs

*To place undue emphasis on retaining an existing resource when making a decision when another unowned resource might be superior.*

Sunk Costs emerged from economics research as an impediment to making sound financial decisions. In the present study 32% of the MLA leader respondents cited Sunk Costs as the second-most selected form of witnessed cognitive bias. In many respects, Sunk Costs resembles Status Quo bias in that both involve resistance to change. While Status Quo bias pertains more to habitual or routine thinking, Sunk Costs relates more to a focus on resources. Sunk costs are expenditures in the past and thereby irrelevant to making a current decision because that expenditures already occurred in the past. The Sunk Cost bias occurs when someone in the present day decides on a matter on the basis of the past expenditure. The resources need not be measurable in literal monetary terms, but can include one's invested time or energy [34].

Several studies have analyzed the likely motivations or external economic forces that lead to Sunk Costs bias [35–39], while one study offers concrete suggestions for mitigation [40].

### Novelty

*The initial fascination and enthusiasm for a new technology or an innovation that does not yet have the needed evidence to support its adoption.*

Novelty bias poses a likely occupational hazard due to our reliance upon new information technology. A total of 28% of the MLA leaders voted for the frequency of their observing others engaged in Novelty bias. Many of our non-HIP colleagues have come to expect us to engage with new technology as unofficial institutional early adapters [41]. New information technology often involves complex relationships with vendors wanting to make large sales so these decisions can be expensive for an institution. Studies have illustrated how positive early reports on new innovations often are countered or at least tempered by subsequent added studies or by more rigorous studies [42–43]. The top three ranked forms of cognitive bias among HIPs, thus far, probably reflect a larger societal tension between the need to innovate with confronting the practicalities of conserving resources and maintaining efficient operations.

### Professionology

*Viewing a situation through the shared perceptions of one's profession rather than by taking a broader perspective. Sometimes known as “Professional Deformation.”*

Professionology might be the oldest forms of cognitive bias recognized by the social sciences, although it has not been extensively studied since its initial identification in 1915. From the outset, it was seen as a distortion that people undergo in the process of their socialization into a specific profession. It derived, in part, from a sense of “exaggerated importance” [Page 31] attached to one's professional roles [44]. Military professionals were portrayed in this study as epitomizing “professional deformation” (as it was once known). Physicians, attorneys, social workers, teachers, nurses, and members of the clergy also were susceptible to Professionology [44]. The implicit sense of a separate if not superior identity seems to reinforce a sense of Professionology in most or possibly all professions [45–55]. Professionology represents a form of the broader and more studied cognitive bias of Ingroup-Outgroup bias [56–60].

The present study revealed that 26% of the MLA leaders identified Professionology as a common form of cognitive bias within our profession. While not much has been researched explicitly on mitigating the bias of Professionology, some limited research has been conducted upon reducing Ingroup Outgroup Bias. One mitigation strategy involves prompting regular interactions between members of the two groups. HIPs have a natural avenue to reduce their Professionology due to their potential for frequent interactions with other health professionals. Framing the two or more groups as members of a broader group can reduce the insularity of any one subgroup within the larger group. Third, encouraging opportunities for friendships or collaborations among members of different groups also might reduce Professionology [61–63]. Explicit efforts to re-classify groups with different categorizations might reduce Ingroup Outgroup Bias [64]. Encouraging members of groups to attempt to be more empathetic toward members of other groups also might help [65–66]. Fostering deeper individual relationships among members of different groups was one promising approach to reducing intergroup bias[67]. One team of researchers has explored the use of ‘science curiosity' as a mitigating strategy for reducing intergroup perceptions. They define science curiosity as an open-minded willingness to engage with surprising information that runs counter to their own attitudes [68–69].

### Authority

*Deferring to an expert or other authority figure disproportionate to the extent of their expertise; or, the range of their authority on the subject*.

Most of us work in hierarchal organizations with clear lines of responsibility for making decisions [70]. This hierarchal context might explain the high ranking in this study of this form of cognitive bias. The practical, ethical, and sometimes legal issues related to abuses of authority are well-known [71–74]. While extreme abuses of authority might lead to authoritarianism [75], more often an authority figure's extension beyond one's range expertise leads to annoyance among those lower in the hierarchy; it also can lead to less efficiency of the organization. While difficult to counter Authority bias, several studies [76–78] have suggested strategies to mitigate as summarized in **Table 3**.

### Worst-Case Scenario

*Emphasizing or exaggerating those possible negative outcomes disproportionate to all possible outcomes*.

Worst-Case Scenario bias was a surprise finding in this study, as it rarely rises to this high a ranking with other surveyed professional populations. The present study produced a 25.5% frequency of mention by HIP leaders. Worst-Case Scenario might be thought of as an extreme form of pessimism [79]. Worst-Case Scenario might represent an historical artifact [80] within this study, prompted by lingering psychological trauma in the US population brought on by the worldwide Covid-19 Pandemic.

Worst-Case Scenario bears a close connection to other similar forms of cognitive bias such as patient Catastrophizing [81–83] And Negativity Bias. [84–87]. The close relationship of the Worst-Case Scenario to Catastrophizing and particularly its to Negativity Effect might lend clues to its mitigation. **Table 3** offers mitigation strategies to Worst Case Scenario bias based on prior research [88–90]. Two studies have cautioned against an absolute rejection of Worst Case Scenario bias due to the possibility that pessimists might have a more realistic view of the situation than others in the group [91].

### Group Think

*Believing in the autonomy of a group, stereotyping of those outside the group, self-censoring, censoring of dissenters, maintaining the illusion of unanimity, and enforcing a group “consensus” viewpoint.*

The present study leveraged the tendency for people to be able to spot cognitive biases in others. Those same cognitive biases are not at all obvious to those observed colleagues. One of the most-often mentioned antidotes to many cognitive biases relies upon the wisdom of the group to detect flaws in individual decision-making processes. Groups are a great way to generate ideas and to spot individual limitations in reasoning that leads to a decision. Singh and Brinster refer to this evolutionary advantage in humans as ‘shared intentionality' (Page 118) in collective action [92].

What happens, though, when the group itself becomes the source of cognitive bias? Group Think was first recognized over 50 years ago when groups of highly intelligent, well-educated US Government officials who were making high-stakes foreign policy decisions succumbed instead to taking dangerous risks [93]. Group Think has been studied in a variety of settings since these early exploratory works. Some of the identified antecedent conditions to Group Think include particular leadership styles, rigid group processes, and certain behaviors [94]. Other factors increasing the likelihood of Group Think include individuals closely aligning their individual identities to the group, attraction to the group itself, and group cohesion. Friendships within a group might exert a mild counterbalance to Group Think [95]. Others seem to have found less supporting evidence for group cohesiveness or certain leadership styles as drivers of Group Think [96]. Several techniques to counter Group Think summarized in **Table 3** have demonstrated some success [97–99]. Group processes oftentimes do not exhibit Group Think. Contexts involving complex variables, emotional competencies, and human relations can generate group processes that definitely can outperform individual efforts [100].

### General Mitigation Strategies

For purposes of efficiency, it might be fruitful to identify general strategies to mitigate our human tendencies to be swayed by all or most of our cognitive biases when making decisions. General mitigation strategies presently are not well-developed and lack sufficient evidence to be much help [101–102]. A few studies offer clues as to how to generally proceed to avoid cognitive biases. Etzioni offers the blunt advice that decision makers should “assume that whatever decisions they render—especially first ones—are wrong and will have to be revised, most likely several times” [103]. Similarly, counterfactual reasoning, the practice of considering one is wrong in a study of 34 nursing students appeared to offset cognitive bias tendencies [104]. Nearly 300 management graduate students reduced their cognitive biases through counterfactual reasoning, provided that these participants were not overconfident of their knowledge of the subject. [105]. One study involved offering a number of plausible outcomes to a decision, rather than just the opposite of what was predicted, to lower cognitive bias scores [106]. Asking decision makers to justify their decisions tended to aid self-reflection to slow any slide into cognitive biases [107]. Skill in scientific reasoning and training in statistics have been found to deter cognitive biases [108–109]. One neuroscientist has suggested that we use a socially-supported environment to make more abstract yet more rational choices more viscerally tangible [110].

### Intergroup Comparisons

It would be interesting to replicate this study involving MLA leaders in several years to compare results. This constellation of cognitive biases resembles and differs from other groups that have taken similar cognitive bias surveys administered by the first author. A seminar of local business leaders in 2008 ranked the following cognitive biases highly: Halo or Horns Effect; Group Think; Anchoring; and, Expectancy Effect. In recent years the first author's second-year medical students have consistently ranked highest Group Think, Confirmation Bias, Authority, and Anchoring forms of cognitive bias.

In recent months the first author has enlisted public health and medical colleagues to replicate this study in their respective professions. Replications could also take place within single HIP workplaces or in different related organizations other than MLA. It would be exciting to use quasi-experimental or randomized controlled trial research designs to test the effectiveness of the aforementioned mitigation strategies.

## LIMITATIONS

Analyzing the representativeness of actual participants in the survey in comparison to the contacted baseline population tends to validate these kinds of surveys. In reference to the peer review process above, these experts will assess representativeness of the participants. For example, if survey respondents only hail from two certain geographic regions of the US, this limitation possibly will modulate the validity of the survey results. Or, as another example, if one type of library is overrepresented, that, too, could modify the interpretation of the results.

On May 15, one participant noticed that the initial list presented to participants did not include the term Group Think. Part 2 of the survey, however, included the term Group Think with its definition in this voting phase. This omission was fixed within 15 minutes at 10am on May 15th by the REDCap Administrator. This omission seems unlikely to have made even a marginal difference given the fact that it did appear with a definition when participants voted.

There are two foreseen deliverables from this study. First, HIPs will benefit in their daily decision-making roles by recognizing the most commonly-encountered forms of cognitive biases. Second, EBP is a framework employed by professionals for making informed decisions. Other than the study in 2007, there are no studies on cognitive biases in decision making contexts for HIPs so this will fill a gap in the research evidence base.

## Data Availability

The full data set for this anonymous survey are available from the Data Appendix at: https://digitalrepository.unm.edu/hslic-publications-papers/97/.

## SUPPLEMENTAL FILES

Appendix A
Note: This appendix document is the *JMLA* supplemental file version of the online appendix referred to in text (see Appendix headline for more information).

## APPENDIX

The following supplementary resources can be found via a single link: https://digitalrepository.unm.edu/hslic-publications-papers/98/

- Supplementary Table One: 2007 Cognitive Bias Summary
- Supplementary Table Two: Participation by MLA Leader Categories
- Detailed Methods Description

## References

[1] Booth A. Barriers and facilitators to evidence-based library and information practice: an international perspective. Perspectives in International Librarianship 2011; 1: 1–15.

[2] Clancy CM, Cronin K. Evidence-based decision making: global evidence, local decisions. Health Aff (Millwood). 2005;24(1):151–162. doi:10.1377/hlthaff.24.1.151 15647226

[3] Eldredge JD. Evidence Based Practice: A Decision-Making Guide for Health Information Professionals. Peer Reviewed, Open Access. Albuquerque, New Mexico: University of New Mexico Health Sciences Library and Informatics Center, 2024. ISBN 979-8-218-34249-4. Available from: <https://www.ncbi.nlm.nih.gov/books/NBK603117/>. Doi: 10.25844/0PWE-9H68 38683908

[4] Sadik MA. The mystery decisions leaders make: Why do leaders make strange decisions when it comes to people? HR Future. 2023;(10):62–64. Accessed April 19, 2024. https://search.ebscohost.com/login.aspx?direct=true&db=bth&AN=172538846&site=ehost-live&scope=site

[5] Lee Y. Evolutionary psychology theory: can I ever let go of my past. In: Appel-Meulenbroek R, Danivska V. A Handbook of Theories on Designing Alignment between People and the Office Environment. (Appel-Meulenbroek R, Danivska V, eds.). Routledge/Taylor & Francis Group; 2021. doi:10.1201/9781003128830

[6] Andrews PW. The psychology of social chess and the evolution of attribution mechanisms: Explaining the fundamental attribution error. Evolution and Human Behavior. 2001;22(1):11–29. doi:10.1016/S1090-5138(00)00059-3 11182572

[7] Haselton MG, Nettle D, Andrews PW. The Evolution of Cognitive Bias. In: Buss DM, ed. The Handbook of Evolutionary Psychology. John Wiley & Sons, Inc.; 2005:724–746.

[8] 10.1002/9780470939376.ch25

[9] Over D. Rationality and the normative description distinction. Koehler DJ Ed. Blackwell Handbook of Judgment and Decision Making. John Wiley & Sons, Incorporated; 2007: 1–18. Accessed March 18, 2025. https://public.ebookcentral.proquest.com/choice/PublicFullRecord.aspx?p=7103359

[10] Dang J. Is there an alternative explanation to the evolutionary account for financial and prosocial biases in favor of attractive individuals? Behav Brain Sci. 2017;40:e25. doi:10.1017/S0140525X16000467 28327235

[11] Lee Y. Behavioral economic theory: masters of deviations, irrationalities, and biases. In: Appel-Meulenbroek R, Danivska V. A Handbook of Theories on Designing Alignment between People and the Office Environment. (Appel-Meulenbroek R, Danivska V, eds.). Routledge/Taylor & Francis Group; 2021. doi:10.1201/9781003128830

[12] Blankenburg Holm D, Drogendijk R, Haq H ul. An attention-based view on managing information processing channels in organizations. Scandinavian Journal of Management. 2020;36(2). doi:10.1016/j.scaman.2020.101106

[13] Leung BTK. Limited cognitive ability and selective information processing. Games and Economic Behavior. 2020;120:345–369. doi:10.1016/j.geb.2020.01.005

[14] Baron J. Thinking and Deciding. Cambridge University Press, 1988: 259–61.

[15] Plous S. The Psychology of Judgment and Decision Making. McGraw-Hill, 1993.

[16] Pronin E, Lin DY, Ross L. The bias blind spot: Perceptions of bias in self versus others. Personality and Social Psychology Bulletin. 2002;28(3):369–381. doi:10.1177/0146167202286008

[17] Pronin E, Hazel L. Humans' bias blind spot and its societal significance. Current Directions in Psychological Science. 2023;32(5):402–409. doi:10.1177/09637214231178745

[18] Pronin E, Gilovich T, Ross L. Objectivity in the Eye of the Beholder: Divergent Perceptions of Bias in Self Versus Others. Psychological Review. 2004;111(3):781–799. doi:10.1037/0033-295X.111.3.781 15250784

[19] Eldredge JD. Cognitive biases as obstacles to effective decision making. Presentation. Fourth International Evidence Based Library and Information Practice Conference (EBLIP4). Durham, NC. 2007.

[20] Helliwell M. Reflections of a Practitioner in an Evidence Based World: 4^th^ International Evidence Based Library & Information Practice Conference. Evidence Based Library and Information Practice, 2(2), 120–122. 10.18438/B8P88R.

[21] Asher M, Hoogland M, Heskett K, Holmes H, Eldredge JD. Making an impact: the new 2024 Medical Library Association Research Agenda, J Med Libr Assoc 2025 Jan; 113(1): 24–30. 39975495 10.5195/jmla.2025.1955 PMC11835027

[22] Baron J. Thinking and Deciding. Cambridge University Press, 1988: 259–61.

[23] Plous S. The Psychology of Judgment and Decision Making. McGraw-Hill, 1993.

[24] Moore DW. Measuring new types of question effects: additive and subtractive. Public Opinion Quarterly 2002; 66: 80–91.

[25] Carp FM. Position effects on interview responses. Journal of Gerontology 1974; 29 (5): 581–87. 4853701 10.1093/geronj/29.5.581

[26] Bostrom N, Ord T. The Reversal Test: Eliminating Status Quo Bias in Applied Ethics*. Ethics. 2006;116(4):656–679. doi:10.1086/505233 17039628

[27] Samuelson W, Zeckhauser R. Status quo bias in decision making. Journal of Risk and Uncertainty. 1988;1(1):7–59. doi:10.1007/BF00055564

[28] Anderson CJ. The Psychology of Doing Nothing: Forms of Decision Avoidance Result from Reason and Emotion. Psychological Bulletin. 2003;129:139–167. 10.1037/0033-2909.129.1.139 12555797

[29] Hosli MO. Power, Connected Coalitions, and Efficiency: Challenges to the Council of the European Union. International Political Science Review / Revue internationale de science politique. 1999;20(4):371–391. 10.1177_019251219902000404.pdf

[30] Geletkanycz MA, Black SS. Bound by the past? Experience-based effects on commitment to the strategic status quo. Journal of Management. 2001; 27(1):3–21. doi:10.1016/S0149-2063(00)00084-2

[31] Camilleri AR, Sah S. Amplification of the status quo bias among physicians making medical decisions. Applied Cognitive Psychology. 2021;35(6):1374–1386. doi:10.1002/acp.3868

[32] Silver WS, Mitchell TR. The status quo tendency in decision making. Organizational Dynamics. 1990; 18(4):34–46. doi:10.1016/0090-2616(90)90055-T

[33] Oschinsky FM, Stelter A, Niehaves B. Cognitive biases in the digital age - How resolving the status quo bias enables public-sector employees to overcome restraint. Government Information Quarterly. 38(4). doi:10.1016/j.giq.2021.101611

[34] Ford JD, Ford LW. Stop Blaming Resistance to Change and Start Using It. Organizational Dynamics. 2010;39(1):24–36. doi:10.1016/j.orgdyn.2009.10.002

[35] Malek N., Acchiardo C.-J. Dismal dating: A student's guide to romance using the economic way of thinking. Journal of Private Enterprise. 2020;35(3):93–108.

[36] Arkes HR, Blumer C. The psychology of sunk cost. Organizational Behavior and Human Decision Processes. 35(1):124–140. doi:10.1016/0749-5978(85)90049-4

[37] Kovács K. The impact of financial and behavioural sunk costs on consumers' choices. Journal of Consumer Marketing. 2024;41(2):213–225. doi:10.1108/JCM-06-2023-6099

[38] Davis LW, Hausman C. Who Will Pay for Legacy Utility Costs? Journal of the Association of Environmental and Resource Economists. 2022;9(6):1047–1085. 10.1086/719793

[39] Jhang J, Lee DC, Park J, Lee J, Kim J. The impact of childhood environments on the sunk-cost fallacy. Psychology & Marketing. 2023;40(3):531–541. doi:10.1002/mar.21750

[40] Rekar P, Pahor M, Perat M. Effect of emotion regulation difficulties on financial decision-making. Journal of Neuroscience, Psychology, and Economics. 2023;16(2):80–93. doi:10.1037/npe0000172

[41] Tamada Y, Tsai T-S. Delegating the decision-making authority to terminate a sequential project. Journal of Economic Behavior and Organization. 2014;99:178–194. doi:10.1016/j.jebo.2014.01.007

[42] Rogers EM. Diffusion of Innovations. 5th ed. Free Press; 2003.

[43] Catalogue of Bias Collaboration. Persaud N, Heneghan C. Novelty Bias. In: Catalogue Of Bias: https://catalogofbias.org/biases/novelty-bias/. Accessed 7 October 2024. 10.1136/bmjebm-2022-112215 37192827

[44] Liang Z, Mao J, Li G. Bias against scientific novelty: A prepublication perspective. Journal of the Association for Information Science and Technology. 2023;74(1):99–114. doi:10.1002/asi.24725

[45] Langerock H. Professionalism: a study in professional deformation. American Journal of Sociology 1915 Jul; 21 (1): 30–44. https://www.jstor.org/stable/2763633

[46] Levin S, Federico CM, Sidanius J, Rabinowitz JL. Social Dominance Orientation and Intergroup Bias: The Legitimation of Favoritism for High-Status Groups. Personality and Social Psychology Bulletin. 2002;28(2):144–157. 10.1177/0146167202282002

[47] Rabow MW, Evans CN, Remen RN. Professional formation and deformation: repression of personal values and qualities in medical education. Fam Med. 2013;45(1):13–18. 23334962

[48] du Toit D. A sociological analysis of the extent and influence of professional socialization on the development of a nursing identity among nursing students at two universities in Brisbane, Australia. J Adv Nurs. 1995;21(1):164–171. doi:10.1046/j.1365-2648.1995.21010164.x 7897070

[49] Horowitz A. On Looking: A Walker's Guide to the Art of Observation. First Scribner hardcover edition. Scribner, an imprint of Simon & Schuster, Inc.; 2013. Accessed December 5, 2024. https://public.ebookcentral.proquest.com/choice/publicfullrecord.aspx?p=5666838

[50] Edwards D. The myth of the neutral reporter. New Statesman 2004 Aug 9: 12–13.

[51] Schudson M. Social Origins of Press Cynicism in Portraying Politics. American Behavioral Scientist. 1999;42(6):998–1008. doi:10.1177/00027649921954714

[52] Bostwick ED, Stocks MH, Wilder WM. Professional Affiliation Bias among CPAs and Attorneys at Publicly Traded US Firms. In: Advances in Accounting Behavioral Research. 2019:121–152. doi:10.1108/S1475-148820190000022007

[53] Witkowski SA. An implicit model for the prediction of managerial effectiveness. Polish Psychological Bulletin. Published online 1996.

[54] MacLean CL, Dror IE. Measuring base-rate bias error in workplace safety investigators. Journal of Safety Research. 2023;84:108–116. doi:10.1016/j.jsr.2022.10.012 36868639

[55] Thomas O, Reimann O. The bias blind spot among HR employees in hiring decisions. German Journal of Human Resource Management. 2023;37(1):5–22. doi:10.1177/23970022221094523

[56] Moskvina NB. The Schoolteacher's Risk of Personality and Professional Deformation. Russian Education and Society. 2006;48(11):74–88.

[57] Moradi Z, Najlerahim A, Macrae CN, Humphreys GW. Attentional saliency and ingroup biases: From society to the brain. Social Neuroscience. 2020;15(3):324–333. doi:10.1080/17470919.2020.1716070 31928322

[58] Wann DL, Grieve FG. Biased Evaluations of In-Group and Out-Group Spectator Behavior at Sporting Events: The Importance of Team Identification and Threats to Social Identity. The Journal of Social Psychology. 2005;145(5):531–545. doi:10.3200/SOCP.145.5.531-546 16201677

[59] Armenta BM, Scheibe S, Stroebe K, Postmes T, Van Yperen NW. Dynamic, not stable: Daily variations in subjective age bias and age group identification predict daily well-being in older workers. Psychology and Aging. 2018;33(4):559–571. doi:10.1037/pag0000263 29902055

[60] Van Bavel JJ, Pereira A. The partisan brain: An identity-based model of political belief. Trends in Cognitive Sciences. 2018;22(3):213–224. doi:10.1016/j.tics.2018.01.004 29475636

[61] Weimer DL. Institutionalizing Neutrally Competent Policy Analysis: Resources for Promoting Objectivity and Balance in Consolidating Democracies. Policy Studies Journal. 2005;33(2):131–146. doi:10.1111/j.1541-0072.2005.00098.x

[62] Gaertner SL, Dovidio JF, Rust MC, et al. Reducing intergroup bias: Elements of intergroup cooperation. Journal of Personality and Social Psychology. 1999;76(3):388–402. doi:10.1037/0022-3514.76.3.388 10101876

[63] Dovidio JF, Love A, Schellhaas FMH, Hewstone M. Reducing intergroup bias through intergroup contact: Twenty years of progress and future directions. Group Processes & Intergroup Relations. 2017;20(5):606–620. doi:10.1177/1368430217712052

[64] Ensari N, Miller N. The out-group must not be so bad after all: The effects of disclosure, typicality, and salience on intergroup bias. Journal of Personality and Social Psychology. 2002;83(2):313–329. doi:10.1037/0022-3514.83.2.313 12150230

[65] Crisp RJ, Hewstone M, Rubin M. Does multiple categorization reduce intergroup bias? Personality and Social Psychology Bulletin. 2001;27(1):76–89. doi:10.1177/0146167201271007 16143674

[66] Prati F, Crisp RJ, Rubini M. 40 years of multiple social categorization: A tool for social inclusivity. European Review of Social Psychology. 2021;32(1):47–87. doi:10.1080/10463283.2020.1830612

[67] Hewstone M, Rubin M, Willis H. Intergroup bias. Annual Review of Psychology. 2002;53(1):575–604. doi:10.1146/annurev.psych.53.100901.135109 11752497

[68] Liebkind K, Haaramo J, Jasinskaja-Lahti I. Effects of contact and personality on intergroup attitudes of different professionals. Journal of Community & Applied Social Psychology. 2000;10(3):171–181. doi:10.1002/1099-1298(200005/06)10:3<171::AID-CASP557>3.0.CO;2-I

[69] Kahan DM, Landrum A, Carpenter K, Helft L, Jamieson KH. Science curiosity and political information processing. Political Psychology. 2017;38(Suppl 1):179–199. doi:10.1111/pops.12396

[70] Motta M, Chapman D, Haglin K, Kahan D. Reducing the administrative demands of the Science Curiosity Scale: A validation study. International Journal of Public Opinion Research. 2021;33(2):215–234. doi:10.1093/ijpor/edz049

[71] Aghion P, Tirole J. Formal and Real Authority in Organizations. Journal of Political Economy. 1997;105(1):1–29. 10.1086/262063

[72] Ross L, Nisbett RE. The Person and the Situation: Perspectives of Social Psychology. Temple University Press; 1991: 52–8.

[73] Milgram S. Obedience to Authority an Experimental View. Harper & Row; 1974.

[74] Hock RR. Forty Studies That Changed Psychology: Explorations into the History Psychological Research. 7th ed. Pearson; 2013: 306–15.

[75] Blass T. The Milgram Paradigm After 35 Years: Some Things We Now Know About Obedience to Authority^1^. Journal of Applied Social Psychology. 1999;29(5):955–978. doi:10.1111/j.1559-1816.1999.tb00134.x

[76] Albright MK, Woodward W. Fascism: A Warning. First edition. Harper, an imprint of HarperCollins Publishers; 2018.

[77] Shi R, Guo C, Gu X. Authority updating: An expert authority evaluation algorithm considering post-evaluation and power indices in social networks. Expert Systems. 2021;38(1). doi:10.1111/exsy.12605

[78] Tarnow E. Towards the Zero Accident Goal: Assisting the First Officer: Monitor and Challenge Captain Errors. Journal of Aviation/Aerospace Education & Research. 2000;10(1):8. DOI: 10.15394/jaaer.2000.1269

[79] Austin JP, Halvorson SAC. Reducing the Expert Halo Effect on Pharmacy and Therapeutics Committees. JAMA. 2019;321(5):453–454. doi:10.1001/jama.2018.20789 30657521

[80] Hey JD. The Economics of Optimism and Pessimism: A D. Kyklos. 1984;37(2):181–205. doi:10.1111/j.1467-6435.1984.tb00748.x (p. 183).

[81] Shadish WR, Cook TD, Campbell DT. Experimental and Quasi-Experimental Designs for Generalized Causal Inference. Houghton Mifflin; 2002: 56; 179;

[82] Sullivan MJL. The Pain Catastrophizing Scale: Development and Validation. Psychological Assessment. 1995;7(4):524–532. DOI:10.1037/1040-3590.7.4.524

[83] Garnefski N, Kraaij V. Cognitive emotion regulation questionnaire - development of a short 18-item version (CERQ-short). Personality and Individual Differences. 2006; 41(6):1045–1053. doi:10.1016/j.paid.2006.04.010

[84] Zhan L, Lin L, Wang X, Sun X, Huang Z, Zhang L. The moderating role of catastrophizing in day-to-day dynamic stress and depressive symptoms. Stress and Health: Journal of the International Society for the Investigation of Stress. 2024;40(4). doi:10.1002/smi.3404 38635165

[85] Kellermann K. The negativity effect and its implications for initial interaction. Communication Monographs. 1984;51(1):37–55. (p. 37). doi:10.1080/03637758409390182

[86] Yang L, Unnava HR. Ambivalence, Selective Exposure, and Negativity Effect. Psychology and Marketing. 2016;33(5):331–343. doi:10.1002/mar.20878

[87] Klein JG. Negativity Effects in Impression Formation: A Test in the Political Arena. Personality and Social Psychology Bulletin. 1991;17(4):412–418. doi:10.1177/0146167291174009

[88] Klein JG, Ahluwalia R. Negativity in the Evaluation of Political Candidates. Journal of Marketing. 2005;69(1):131–142. 10.1509/jmkg.69.1.131.5550

[89] Fracalanza K, Raila H, Rodriguez CI. Could written imaginal exposure be helpful for hoarding disorder? A case series. Journal of Obsessive-Compulsive and Related Disorders. 2021; 29: 1–5. doi:10.1016/j.jocrd.2021.100637

[90] Goodwin P, Gönül S, Önkal D, Kocabıyıkoğlu A, Göğüş CI. Contrast effects in judgmental forecasting when assessing the implications of worst and best case scenarios. Journal of Behavioral Decision Making. 2019;32(5):536–549. doi:10.1002/bdm.2130

[91] Wood S, Kisley MA. The negativity bias is eliminated in older adults: age-related reduction in event-related brain potentials associated with evaluative categorization. Psychology and aging. 2006;21(4):815–820. DOI: 10.1037/0882-7974.21.4.815 17201501

[92] Sonoda A. Optimistic bias and pessimistic realism in judgments of contingency with aversive or rewarding outcomes. Psychological reports. 2002;91(2):445–456. 12416837 10.2466/pr0.2002.91.2.445

[93] Singh R, Brinster KN. Fighting Fake News: The Cognitive Factors Impeding Political Information Literacy. In: Libraries and the Global Retreat of Democracy: Confronting Polarization, Misinformation, and Suppression. 2021:109–131. doi:10.1108/S0065-283020210000050005

[94] Janis IL. Groupthink and group dynamics: a social psychological analysis of defective policy decisions. Policy Studies Journal. 1973 Sep;2(1):19–25. doi:10.1111/j.1541-0072.1973.tb00117.x

[95] Schafer M, Crichlow S. Antecedents of Groupthink: A Quantitative Study. Journal of Conflict Resolution. 1996;40(3):415–435. doi:10.1177/0022002796040003002

[96] Hogg MA, Hains SC. Friendship and group identification: a new look at the role of cohesiveness in groupthink. European Journal of Social Psychology. 1998;28(3):323–341. doi:10.1002/(SICI)1099-0992(199805/06)28:3<323::AID-EJSP854>3.0.CO;2-Y

[97] Kerr NL, Tindale RS. Group performance and decision making. Annual review of psychology. 2004;55:623–655. 10.1146/annurev.psych.55.090902.142009 14744229

[98] Janis IL. Groupthink: psychological studies of policy decisions and fiascoes. Boston: Houghton Mifflin Company, 1983: 260–76.

[99] Hodson G, Sorrentino RM. Groupthink and uncertainty orientation: Personality differences in reactivity to the group situation. Group Dynamics: Theory, Research, and Practice. 1997;1(2):144–155. doi:10.1037/1089-2699.1.2.144

[100] Lee CE, Martin J. Obama warns against White House ‘groupthink.’ Politico December 1,2008. Available from: <https://www.politico.com/story/2008/12/obama-warns-against-wh-groupthink-016076>. Accessed 8 December 2024.

[101] De Villiers R, Hankin R, Woodside AG. Making decisions well and badly: how stakeholders' discussions influence executives' decision confidence and competence. In: Woodside AG. Making Tough Decisions Well and Badly: Framing, Deciding, Implementing, Assessing. Emerald; 2016. Accessed December 6, 2024. http://www.dawsonera.com/depp/reader/protected/external/AbstractView/S9781786351197

[102] Korteling JEH, Gerritsma JYJ, Toet A. Retention and Transfer of Cognitive Bias Mitigation Interventions: A Systematic Literature Study. Frontiers in psychology. 2021;12:629354. doi:10.3389/fpsyg.2021.629354 34456780 PMC8397507

[103] Shlonsky A, Featherston R, Galvin KL, et al. Interventions to Mitigate Cognitive Biases in the Decision Making of Eye Care Professionals: A Systematic Review. Optom Vis Sci. 2019;96(11):818–824. doi:10.1097/OPX.0000000000001445 31664015

[104] Etzioni A. Humble Decision-Making Theory. Public Management Review. 2014;16(5):611–619. [Page 612]. doi:10.1080/14719037.2013.875392

[105] Flannelly LT, Flannelly KJ. Reducing People's Judgment Bias About Their Level of Knowledge. The Psychological record. 2000;50(3):587. DOI: 10.1007/BF03395373

[106] Hoch SJ. Counterfactual reasoning and accuracy in predicting personal events. Journal of Experimental Psychology: Learning, Memory, and Cognition. 1985;11(4):719–731. doi:10.1037/0278-7393.11.1-4.719

[107] Hirt ER, Markman KD. Multiple explanation: A consider-an-alternative strategy for debiasing judgments. Journal of Personality and Social Psychology. 1995;69(6):1069–1086. doi:10.1037/0022-3514.69.6.1069

[108] Isler O, Yilmaz O, Dogruyol B. Activating reflective thinking with decision justification and debiasing training. Judgment and Decision Making. 2020;15(6):926–938. doi:10.1017/S1930297500008147

[109] Cavojová V, Šrol J, Jurkovic M. Why Should We Try to Think Like Scientists? Scientific Reasoning and Susceptibility to Epistemically Suspect Beliefs and Cognitive Biases. Applied Cognitive Psychology. 2020;34(1):85–95.

[110] Fong GT. The Effects of Statistical Training on Thinking about Everyday Problems. Cognitive Psychology. 1986;18(3):253–292

[111] Falk E. The science of making better decisions. New York Times. 2025 July 6; Opinion: 10.

