# Supplementary material for: Cognitive biases as interrupters in evidence based practice decision-making
Source: J Med Libr Assoc. 2025 Oct 23;113(4):269–80. doi: 10.5195/jmla.2025.2209 (PMC12604062; doi:10.5195/jmla.2025.2209)
Supplement: Supplementary file 1 — Appendix A [file jmla-113-4-269-s01.docx]

**Cognitive Biases as Interrupters in Evidence Based Practice Decision-Making**

Journal of the Medical Library Association

**Online Appendix**

**Supplementary Table 1**

**Most Commonly Observed Cognitive Biases among HIPs in 2007**

| **Cognitive Bias Type** | **Numbers** | **Percentages** |
| --- | --- | --- |
| Professionology (Professional Deformation) | 44 | 11% |
| Status Quo | 42 | 11% |
| Authority | 28 | 7% |
| Anchoring | 27 | 7% |
| Groupthink | 26 | 7% |
| Halo or Horns Effect | 25 | 6% |

Study conducted with 135 respondents

Sources:

Eldredge JD. Cognitive biases as obstacles to effective decision making in EBLIP. Contributed Paper. Fourth International Evidence Based Library and Information Practice Conference. Chapel Hill, NC. May 7, 2007.

Eldredge JD. Evidence-based practice. In: *Introduction to health sciences librarianship*. Edited by Sandra Wood. Binghamton, NY: Haworth Press, 2008: 245-69.

**Supplementary Table 2**

**Leader Status of Respondents**

| **Status** | **Respondents** |
| --- | --- |
| MLA Caucus | 58 (38.9%) |
| MLA Chapter Elected Leader | 48 (32.2%) |
| MLA National Level Committee | 27 (18.1) |
| Other | 14 (9.4%) |
| MLA Officer, Board Member, or Editor | 9 (6.0%) |
| *Total* | *149 respondents* |

**Detailed Methods Description**

*Creating the Inventory*

Assembling an inventory of cognitive biases most likely to be found among health information professionals began with a series of iterative literature searches in multiple databases. The literature searches were intended to provide updated research findings on cognitive biases since the 2007 study. A major task for these literature searches became determining which cognitive biases might apply to decision making specifically for Health Information Professionals (HIPs). Some cognitive biases, for example, relate to the justice system and have marginal relevance to decision making in our profession. Some clinical environments, moreover, involve only a few cognitive biases relevant to our profession. The goal was to compile a master list of *observable* cognitive biases that relate most directly to decision making in the health information professions.

The literature searches were intended to identify cognitive biases potentially relevant to decisions made by HIPs. Two disorienting aspects uncovered during the literature searches were the lack of standard terminology for some cognitive biases and the minor overlaps in the described behavioral patterns. Clinical researchers similarly have pointed to the dire need for standardized language to consistently describe cognitive biases in clinical medicine. [1]

*Literature Searches*

The PsycINFO database contained most of the potentially relevant research literature on cognitive biases. Using the APA Thesaurus term “Cognitive Bias” retrieved 9,771 references, which proved to be an unworkable number. Starting on January 2, 2024 the first author started to test search strategies for the desired sensitivity and specificity for this project. On January 18, 2024 the first author finally settled on using the Thesaurus terms “Cognitive bias” AND “Decision Making” as major concepts for the years 2013-2023. This search retrieved 301 article references. Reading the abstracts using a fairly long list of exclusion criteria led to the selection of only 39 article references. These exclusion criteria pertained to highly rarified situations, memory tests, gaming, judicial decisions, abstract experiments, financial market speculations, clinical emergencies, law enforcement, or student populations in academic settings. Only about 30 of these references, upon examining the articles themselves, were even tangentially suitable for the project. Filtering the same PsycINFO strategy to only books and book chapters offered a broader view of cognitive biases. The Ramos book, cited below, provided an immensely helpful inventory of cognitive biases.

A series of iterative PubMed searches during January-March 2024 led to the most promising search strategy on April 1, 2024 combining a controlled vocabulary and linked phrase approach: Decision making [Mesh] AND “cognitive bias*” that produced 440 references with no applied filters. Most retrieved references were excluded because they pertained to: patients exhibiting cognitive biases in a diseased state such as mental illness or addiction; faulty patient self-diagnosis; cognitive biases among clinicians interfering with their making accurate diagnoses; specific situations with providers managing a disease yet complicated by cognitive biases; basic science investigations; commentaries or narrative reviews that discussed various studies on cognitive biases; or, research studies involving rarefied situations. The low number of 36 possibly relevant references in PubMed resulted in further limiting the search to the years 2018-2024. A closer examination of the abstracts led to zero (0) references potentially relevant to creation of the survey instrument, although seven of the references pertained to strategies for mitigating cognitive bias. These articles reporting on mitigation strategies were retained for the Discussion section of this article for their potential use in overcoming certain cognitive biases in the future.

The Public Affairs Information Service (PAIS) database lacked a strong controlled vocabulary like PsycInfo or PubMed so after some trial-and-error approaches during early January 2024, the first author retrieved 755 references using the controlled vocabulary term “Decision making” coupled to limits for 2013-2024 and for only scholarly articles. There were many false positives due to the sensitivity of this search. By examining the abstracts and the actual text of some articles the list eventually was winnowed down to 22 references after implementing the exclusion criteria used for the PsycINFO searches. At that juncture, about 10 of these references seemed like they might be potentially useful in interpreting study results.

A search in the Business Source Complete database on February 8, 2024 using the phrase “cognitive bias” and the controlled vocabulary descriptor “Decision making” supplemented with searches involving the names of specific cognitive biases only produced 5 potentially useful references.

The keyword adjacent search of “cognitive bias*” in Library, Information Science & Technology Abstracts (LISTA) for the years 2013 to 2024 on March 10, 2024 produced 172 references. Only 21 of these references had any initial potential relevance to constructing an inventory of cognitive biases in the survey instrument. Several articles examined cognitive biases in the information retrieval process, [2-4] but this was a specialized form of decision-making that was not unique to HIPs. Other articles on cognitive bias related to its possible role in spreading misinformation. [5-8] All 21 references were excluded due to their low utility for building an inventory of cognitive biases relevant to health information professionals.

The literature searches provided some articles of potential value later in interpreting the results of this study. Ultimately, the literature searches provided no direct guidance on constructing an inventory of cognitive biases related to decisions made by HIPs. Another approach was needed for developing the survey.

*Developing the Survey*

Verónica Juȧrez Ramos authored the 2019 book *Analyzing the Role of Cognitive Biases in the Decision-Making Process*. [9] It proved to be the most helpful single comprehensive resource for this project due to her descriptions of 27 “important” forms of cognitive bias related to decision making followed by an extensive inventory totaling 177 forms of cognitive bias with even a tangential possible connection with decision-making. In the later chapters she introduces several more forms of cognitive biases. Ramos also provides some overall coherence to the subject for the newcomer to the field and pulls together many aspects of cognitive bias from her extensive literature searches. The first author cross-checked inventories of cognitive biases [10-13] found in other less comprehensive sources to ensure including a large range of pertinent forms of cognitive bias.

The Ramos book does have a number of unfortunate syntaxial, grammatical, and reference errors. References sometimes are made in the text without the corresponding full references listed at the end of the chapters. By taking in a broad sweep of the subject, the book focuses on forms of cognitive bias irrelevant to purposes of decision making among HIPs. At times, she uses different words or phrases other than either more established terms or the terms used by other more recent authors to describe the same or nearly the same cognitive bias phenomena. The cognitive bias form known as Groupthink that appeared in the 2007 Eldredge study has a number of near equivalents in Ramos’ book inventory: Bandwagon, Herd Instinct, In Group Bias, Outgroup Homogeneity, Shared Information, and Ultimate Attribution Error. The 2007 survey included the other well-researched cognitive bias Status Quo Bias that has near equivalents in the Ramos book: Conservatism, Endowment, Illusion of Truth, Loss Aversion, Risk Aversion, and System Justification. For purposes of this research study, however, the Ramos book offered the most viable comprehensive inventory with a sufficient number of terms linked to relevant research studies. The authors credit Ramos for undertaking such a challenging task and for developing a viable and comprehensive inventory for the present study.

This project sought to understand better the kinds of cognitive biases that HIPs might exhibit in individual or in group situations. **Methods Table 1** lists cognitive biases that do not traverse both individual *and* group contexts that instead fall into one or the other distinct category. This study has excluded those forms of cognitive bias in **Methods** **Table 1** for these reasons. The two exceptions were GroupThink and Professionology because these two forms involve a group perspective becoming internalized by individual group members.

**Methods Table 1 Cognitive Biases that are Solely Either Individual- or Group-Oriented**

| **Individual** | **Group (Social)** |
| --- | --- |
| Above-Average Effect  Affect Heuristic  Attentional Bias  Bias Against Evidence Confirmatory  Bias Blind Spot  Congruence Bias  Cryptomnesia  Defensive Attribution  Dunning-Kruger  Duration Neglect  Egocentric Bias  Essentialism  False Consensus  Moral Licensing of Credential  Optimism Effect  Overconfidence Bias  Projection  Restraint  Risk Compensation (Peltzman Effect)  Self-Relevance  Self-Serving  Spotlight  Subadditivity  Subjective Validation  Trait Ascription  Worse-Than-Average | Actor-Observer Bias  Availability Cascade  Cross-Race Effect  Experimenter Expectancy  Group Attribution  Rhyme-as-Reason  Social Desirability |

Consumer decisions that also might overlap with the excluded financial cognitive biases in **Methods Table 2** includes Denomination, Frequency, and Unit Bias. There are other contexts involving cognitive bias that do not relate to the current project. In politics the cognitive biases Availability Cascade, Hostile Media Effect, and Zero-Sum Heuristic have little bearing on HIPs’ decisions. Numerical literacy (Numeracy) situations relating to financial risk that have been excluded include: Illusion of Validity, and Insensitivity to Sample Size. Cognitive biases excluded in the present study that occur in learning situations include: Generation Effect, Ikea Effect, and Verbatim Effect.

**Methods Table 2: Other Forms of Cognitive Bias in Decisions *Excluded* from this Project**

| **Financial** | **Memory** | **Gaming** |
| --- | --- | --- |
| Disposition  Endowment  Extrinsic Incentives  Hyperbolic Discounting  Loss Aversion  Money Illusions  Naïve Cynicism  Not Invented Here  Scope Insensitivity | Bizarreness  Change Bias  Context Effect  Fading Effect  False Memory  Focusing Effect  Google Effect  Humor Effect  Lag Effect  Mere Exposure  Misinformation  Modality  Mood Congruency  Negativity Bias  Omission  Part-List Cueing  Persistence  Recency Effect  Rosy Retrospection  Serial Position  Source Confusion  Spacing Effect  Suffix Effect  Suggestibility  Telescoping  Testing Effect  Tip of the Tongue  Von Restoroff  Zelgarnik | Ambiguity  Base Rate Fallacy  Disregard of Regression  Forward Bias  Hot Hand  List Length  Zero Risk |

Importantly, the aforementioned lists contain terms that have specific definitions that differ from more common usage of the same terms.

*Piloting the Survey Instrument*

The penultimate form of the survey included 24 forms of cognitive bias. The authors determined that 24 choices with accompanying brief definitions would present the maximum cognitive load for most participants. To ensure coherence and understandability of the survey the first author recruited 11 colleagues to pilot the survey in Word form. Volunteers responding to the first author’s request cast their individual votes for up to five (5) forms of cognitive bias in the pilot form of the survey. Aside from some minor issues of sequential flow, the colleagues piloting the survey found the definitions to be clearly-understood and the survey was easy to navigate. Several colleagues volunteered to pilot the actual REDCap (**R**esearch **E**lectronic **D**ata **Cap**ture) survey, mostly for functionality of the interface. The authors received the final IRB approval (24-168) on April 11, 2024.

File: Cognitive Bias 2024 Manuscript Appendices 20250701

**REFERENCES**

1.Satya-Murti S, Lockhart JJ. Needed: consensus and classification for terms used in cognitive, forensic and clinical bias discussions. *Forensic Sci Int*. 2018;293:e10-e11. doi:10.1016/j.forsciint.2018.09.028

2.Gomroki G, Behzadi H, Fattahi R, Fadardi JS. The Role of Demographic Variables in Frequency of Cognitive Biases in Information Retrieval. *Iranian Journal of Information Processing & Management*. 2020;36(1):127-153. Accessed July 11, 2024. https://search.ebscohost.com/login.aspx?direct=true&db=lxh&AN=147005501&site=ehost-live&scope=site

3.Gomroki G, Behzadi H, Fattahi R, Salehi Fadardi J. Identifying effective cognitive biases in information retrieval. *Journal of Information Science*. 2023;49(2):348-358. doi:10.1177/01655515211001777

4.Lau AY, Coiera EW. Do people experience cognitive biases while searching for information? *Journal of the American Medical Informatics Association*. 2007;14(5):599-608. doi:10.1197/jamia.m2411

5.Soprano M, Roitero K, La Barbera D, et al. Cognitive Biases in Fact-Checking and Their Countermeasures: A Review. *Information Processing & Management*. 2024;61(3):N.PAG. doi:10.1016/j.ipm.2024.103672

6.Gwebu KL, Wang J, Zifla E. Can warnings curb the spread of fake news? The interplay between warning, trust and confirmation bias. *Behaviour & Information Technology*. 2022;41(16):3552-3573. doi:10.1080/0144929X.2021.2002932

7.Savolainen R. What drives people to prefer health-related misinformation? The viewpoint of motivated reasoning. *Information Research*. 2022;27(2):N.PAG. Accessed July 11, 2024. https://search.ebscohost.com/login.aspx?direct=true&db=lxh&AN=157461328&site=ehost-live&scope=site

8.Singh R, Brinster KN. Fighting Fake News: The Cognitive Factors Impeding Political Information Literacy. *Advances in Librarianship*. 2021;50:109-131. doi:10.1108/S0065-283020210000050005

9.Ramos VJ. Analyzing the Role of Cognitive Biases in the Decision-Making Process. Information Science Reference; 2019. Accessed March 28, 2024. doi=10.4018/978-1-5225-2978-1.

# 10.Pronin E, Hazel L. Humans’ bias blind spot and its societal significance. Appendix A. Current Directions in Psychological Science 2023; 32(5): 402-9. https://doi.org/10.1177/09637214231178745

11.Korteling JEH, Gerritsma JYJ, Toet A. Retention and Transfer of Cognitive Bias Mitigation Interventions: A Systematic Literature Study. *Front Psychol*. 2021;12:629354. Published 2021 Aug 12. doi:10.3389/fpsyg.2021.629354

12.Dickey CC, Thomas C, Feroze U, Nakshabandi F, Cannon B. Cognitive Demands and Bias: Challenges Facing Clinical Competency Committees. *J Grad Med Educ*. 2017;9(2):162-164. doi:10.4300/JGME-D-16-00411.1

13.Featherston R, Downie LE, Vogel AP, Galvin KL. Decision making biases in the allied health professions: A systematic scoping review. *PLoS One*. 2020;15(10):e0240716. Published 2020 Oct 20. doi:10.1371/journal.pone.0240716

File: Cognitive Bias 2024 Manuscript Appendices 20250701
